# Sustainable Primary Cell Banking for Topical Compound Cytotoxicity Assays: Protocol Validation on Novel Biocides and Antifungals for Optimized Burn Wound Care

**DOI:** 10.3390/ebj5030024

**Published:** 2024-08-06

**Authors:** Zhifeng Liao, Nicolas Laurent, Nathalie Hirt-Burri, Corinne Scaletta, Philippe Abdel-Sayed, Wassim Raffoul, Shengkang Luo, Damian J. Krysan, Alexis Laurent, Lee Ann Applegate

**Affiliations:** 1 Plastic, Reconstructive and Hand Surgery Service, Lausanne University Hospital, University of Lausanne, CH-1011 Lausanne, Switzerland; liao.zhifeng@unil.ch (Z.L.); nicolas.laurent@chuv.ch (N.L.); nathalie.burri@chuv.ch (N.H.-B.); corinne.scaletta@chuv.ch (C.S.); philippe.abdel-sayed@chuv.ch (P.A.-S.); 2 Lausanne Burn Center, Lausanne University Hospital, University of Lausanne, CH-1011 Lausanne, Switzerland; 3 STI School of Engineering, Federal Polytechnical School of Lausanne, CH-1015 Lausanne, Switzerland; 4 Plastic and Reconstructive Surgery, Ensemble Hospitalier de la Côte, CH-1110 Morges, Switzerland; wassim.raffoul@ehc.vd.ch; 5 Plastic and Reconstructive Surgery, Guangdong Second Provential General Hospital, Guangzhou 510317, China; luoshk@gd2h.org.cn; 6 Stead Family Department of Pediatrics, Carver College of Medicine, Stead Family Children’s Hospital, University of Iowa, Iowa City, IA 52242, USA; damian-krysan@uiowa.edu; 7 Manufacturing Department, TEC-PHARMA SA, CH-1038 Bercher, Switzerland; 8 Manufacturing Department, LAM Biotechnologies SA, CH-1066 Epalinges, Switzerland; 9 Center for Applied Biotechnology and Molecular Medicine, University of Zurich, CH-8057 Zurich, Switzerland; 10 Oxford OSCAR Suzhou Center, Oxford University, Suzhou 215123, China

**Keywords:** adipose-derived stem cells, antifungals, biocides, burn wounds, chlorhexidine, cytotoxicity assays, dermal fibroblasts, disinfectants, hypochlorous acid, primary cell banking

## Abstract

Thorough biological safety testing of topical therapeutic compounds and antimicrobials is a critical prerequisite for appropriate cutaneous wound care. Increasing pathogen resistance rates to traditional antibiotics and antifungals are driving the development and registration of novel chemical entities. Although they are notably useful for animal testing reduction, the gold standard in vitro cytotoxicity assays in continuous cell lines (HaCaT keratinocytes, 3T3 fibroblasts) may be discussed from a translational relevance standpoint. The aim of this study was thus to establish and validate a sustainable primary cell banking model with a view to performing optimized in vitro cytotoxicity assay development. Primary dermal fibroblasts and adipose-derived stem cell (ASC) types were established from four infant polydactyly sources. A multi-tiered primary cell banking model was then applied to prepare highly sustainable and standardized dermal fibroblast and ASC working cell banks (WCBs), potentially allowing for millions of biological assays to be performed. The obtained cellular materials were then validated for use in cytotoxicity assays through in vitro biosafety testing of topical antiseptics (chlorhexidine, hypochlorous acid) and an antifungal compound (AR-12) of interest for optimized burn wound care. The experimental results confirmed that IC_50_ values were comparable between cytotoxicity assays, which were performed with cell lines and with primary cells. The results also showed that hypochlorous acid (HOCl) displayed an enhanced toxicological profile as compared to the gold standard chlorhexidine (CLX). Generally, this study demonstrated that highly sustainable primary cell sources may be established and applied for consistent topical compound biological safety assessments with enhanced translational relevance. Overall, the study underscored the safety-oriented interest of functionally benchmarking the products that are applied on burn patient wounds for the global enhancement of burn care quality.

## 1. Introduction

Infectious complications of cutaneous burn wounds affect patient quality-of-life and incur high financial burdens [1,2,3]. In clinical practice, the preventive cutaneous application of topical antiseptics is essential during burn wound treatment [4]. However, systematic and empirical antibiotic and antiseptic product use is debated, with notable concerns around topical compound cytotoxicity and the potential for induced antibiotic/antifungal resistance [4,5]. Furthermore, while the functional characteristics of antiseptics are well-described, comparative compound topical cytotoxicity data are scarcely reported [6,7]. With the latter being time- and dose-dependent, they should be extensively characterized for therapeutic preparations that are clinically used on a large scale [7]. In burn patient care, the gold standard in topical disinfectants (i.e., in various concentrations and product formulations) was chlorhexidine (CLX) for many years [8]. Although the existing in vivo data are not clear, the in vitro cytotoxicity of CLX is well documented and shows potential to hamper burn wound healing [4,9,10]. Thus, CLX is no longer primarily recommended for open wound cleansing protocols, as new and safer substances are commercially available [6,7,8,9,10]. 

Specifically, emerging resistant bacteria and increasing fungal infection rates in burn patients warrant the screening of alternatives to CLX, with a focus on functional molecules and low-cytotoxicity compounds [6,7,8]. Notably, recommendations are emerging for the topical use of hypochlorous acid (HOCl), which is commercially available mainly for surface cleaning and disinfection [11,12,13]. As a new chemical entity, this compound must be assessed in terms of biological safety (e.g., ISO 10993 norm) prior to introduction as a biocidal device on the EU and Swiss markets for human topical use [14,15,16]. Therein, cellular models for in vitro cytotoxicity testing (i.e., various product topical formulations) using primary human cells could be used as alternatives to assays based on immortalized cell lines for enhanced data relevance in burn care settings [6,7,17,18,19]. Such assays are essential in compound biosafety testing, thereby justifying the necessity for robust in vitro methods that are compatible with high-throughput screening, using relevant target cell sources [18,20]. 

Of note, in vitro cellular models are increasingly used in drug screening and toxicity assays, as they are recognized as valid alternatives to animal models [21]. Therein, the majority of the cellular models that are used for cytotoxicity assays employ transformed (i.e., immortalized) cell sources (i.e., human and animal cell lines, e.g., 3T3, CHO, HEK, HaCaT cells) [22,23,24]. The latter are key components for the in vitro assessment of skin damage and loss of integrity potentially caused by ingredients in cosmetics, medical devices, or pharmaceutical compounds [6,16,25,26]. However, notable disadvantages of cell lines may include high costs, variability, and genetic instability (i.e., due to their tumorigenic proliferative characteristics). Notwithstanding, the main limit of this kind of cell source is that they are not relevant from a species point-of-view (i.e., mouse, hamster cells, etc.) and that they are not fully physiologically relevant to what is occurring in vivo [6,18,22]. Additionally, cell type-specific behaviors may complexify the comparability and the interpretation of in vitro cytotoxicity results. Thus, it is now of interest to focus on alternative cellular models that could be developed using stem cells or primary cells, with an emphasis on finding consistent, sustainable, and standardizable cell sources [18,19,27]. Furthermore, such models should work as efficiently as those recommended by the OECD for cellular toxicity screening, albeit with more physiological relevance and increased sensitivity. Specifically, for regulatory acceptance, an in vitro cell-based assay must be reproductible, reliable, and relevant [14,15,16,28].

In particular, adipose-derived stem cells (ASCs) have been clinically applied in regenerative therapies due to their properties of self-renewal or their ease of availability and could therefore be of interest for in vitro cytotoxicity assays [19,29,30]. These cells may contribute to improving drug safety for patients, reduce experimental animal use, reduce preclinical screening costs, and reduce pharmaceutical drug substance attrition rates. Notwithstanding, the dedifferentiation potential of ASCs, their variability, low cell source sustainability, and the use of many reagents in culture systems to assure their stability make them more difficult to standardize than primary cells that are readily and fully differentiated [27,29]. Indeed, primary cells from specific tissues are considered as a better source for standardized in vitro assays. While primary cells cannot be cultured indefinitely due to the in vitro onset of replicative senescence, it is possible (i.e., with optimized multi-tiered cell banking procedures) to obtain highly sustainable supplies of cells (e.g., MRC-5 and WI-38 lung fibroblast cell types) [31]. Therefore, a master cell bank (MCB) of primary cells could constitute a reproductible and dependable source of identical progeny cell cultures for standardized assays [31].

Importantly, the widespread availability of primary cell starting materials and the ease of their culture methods may aid in providing robust datasets with enhanced physiological relevance. Therein, juvenile surgical waste tissues (e.g., polydactyly digits) bare high potential for extensive primary cell biobanking [32,33]. Notably, dermal fibroblasts (i.e., differentiated cell type) and ASCs (i.e., phenotypically plastic cell type) may be derived from polydactyly donors for sustainable primary cell source establishment. Therefore, the aim of this study was to experimentally establish and validate a polydactyly-derived primary cell banking model for optimized cytotoxicity assay development in the context of burn care. The primary hypothesis of the study was that extensive cryopreserved cell stocks can be established for polydactyly-derived primary dermal fibroblasts and ASCs with a view to performing standardized in vitro cytotoxicity assessments. The secondary hypothesis of the study was that these primary cells can be used for in vitro antiseptic and antifungal product biosafety assessments, similarly to cell line-based models but with enhanced biological relevance. Overall, the present study underscored the need to continuously enhance the safety and quality of products that are applied in burn patient care, which may be achieved via the optimization of preclinical product assessment and benchmarking methodologies. 

## 2. Materials and Methods

### 2.1. Primary Dermal Fibroblast and ASC Source Establishment

Dermal fibroblasts and ASCs were obtained by primary tissue culture and serial cell expansion from the dorsal skin and the sub-cutaneous fat tissue from ablated extra fingers. The procured human tissues were originally from polydactyly patients (i.e., 1, 2, 3.5, and 7 days of age at the time of surgery, respectively) and were registered in the CHUV-DAL Department Biobank under an ethics protocol approval and following the applicable biobanking directive (BB_029_DAL). Overall, four primary dermal fibroblast cell types (i.e., PSK-1, PSK-2, PSK-3, PSK-4) and four ASC types (i.e., PASC-1, PASC-2, PASC-3, PASC-4) were established from the available biological starting materials (i.e., dermis and hypodermal fat, respectively). Cryopreserved HaCaT immortalized human keratinocytes (ATCC, Manassas, VA, USA) were used as cell line controls in the cytotoxicity experiments.

The procured tissues (i.e., integral digits) were transported from the operating room to the laboratory in phosphate-buffered saline (PBS; Bichsel, Unterseen, Switzerland) at ambient temperature. The samples were washed three times for 15 min in PBS supplemented with 1% penicillin-streptomycin (ThermoFisher Scientific, Waltham, MA, USA). Dorsal skin flaps (i.e., ~0.5 cm^2^) including both the epidermis and the dermis were then aseptically dissected into small fragments and were placed in 10 cm diameter tissue culture dishes (Falcon, Durham, NC, USA) using an explant method. The tissue fragments were cultured in complete fibroblast medium (CfM), which was composed of Dulbecco’s modified Eagle medium (DMEM; Life Technologies, Carlsbad, CA, USA) with 25 mM dextrose and 1 mM sodium pyruvate, supplemented with 1% L-glutamine (Life Technologies, Carlsbad, CA, USA) and with 10% fetal bovine serum (FBS; Invitrogen, Carlsbad, CA, USA). The CfM contained no antibiotic supplementation. The hypodermal adipose tissues were processed in the same way for primary culturing of ASCs, with 5% human platelet lysate (HPL; BioLife Solutions, Bothell, WA, USA) instead of FBS (i.e., complete ASC medium; CAM).

For primary cell isolation, the culture dishes were placed in humidified incubators at 37 °C under 5% CO_2_. The CfM was exchanged every three days and the tissue explants were monitored by microscopy. Once the emitting cells reached sufficient confluency levels (i.e., 80–90%, in approximately 10 days), the adherent cells were detached with TrypLE™ (Life Technologies, Carlsbad, CA, USA) and were seeded at 2000 cells/cm^2^ in cell culture T flasks (75 cm^2^; TPP, Trasadingen, Switzerland). The subcultures were expanded to confluency in CfM, which was replaced every three to four days. Confirmation of cell adhesion, cell proliferation, and absence of contamination was iteratively assessed by microscope examination. At confluency (i.e., 90–100%), the adherent cells were rinsed once with PBS (Bichsel, Unterseen, Switzerland) and were harvested with TrypLE™ (Life Technologies, Carlsbad, CA, USA). The cell suspension was then centrifuged for 10 min at 230× *g* at ambient temperature, rinsed with PBS, and enumerated using a Neubauer hemocytometer (NanoEnTek, Seoul, Republic of Korea) and Trypan blue exclusion dye (Corning, Corning, NY, USA).

### 2.2. Primary Cell Subcultures and Tiered Biobanking Model Establishment

The obtained primary cell populations were cryopreserved in 50–100 vials (i.e., each containing 10^6^ cells) at passage level 1. The obtained material lots were defined as the master cell banks (MCBs) and were stored in the liquid nitrogen vapor phase. Through serial subculturing, tier-1 working cell banks (WCB I) at passage level 2 and tier-2 WCB (WCB II) at passage level 3 were derived from the MCBs. All WCB lots were composed of 100–200 vials (i.e., each containing 10^6^ cells). Appropriate in-process controls and post-process controls were performed during the cell expansions and following cryopreserved vial thawing to monitor the quality level of the materials. For all subsequent in vitro cytotoxicity assays, WCB II vials were thawed and the cells were expanded before use, which occurred between passage levels 4 and 14 for the purpose of this study.

### 2.3. Primary Cell Proliferation Assessment and End-of-Passage Determination

In order to experimentally validate the retained primary cell banking model in terms of sustainability (i.e., number of cells available/cell type), serial subcultures of WCB II vials were performed quantitatively for selected donors (i.e., PSK-1/PASC-1 and PSK-2/PASC-2) and qualitatively for all cultures, as all primary cell types displayed excellent cell proliferation capacities at early passages. Therefore, the cells were serially expanded for the purpose of identifying the “Hayflick limit”, which can be associated with an end-of-passage (EOP) cell banking potential. Specifically, the EOP is the higher limit of the cellular proliferative state (i.e., maximal passage level) at which the primary cells should be used for in vitro experimentation. The EOP can be determined by monitoring the cell proliferation curves over time while using consistent manufacturing technical specifications (e.g., cell seeding density, expansion timeframes). 

All of the considered primary cell types included for the quantitative in vitro lifespan assessments were initiated and seeded at 1500 cells/cm^2^ in T25 culture flasks (TPP, Trasadingen, Switzerland) in triplicate. The cultures were maintained as described hereabove for 7 days. Following cell harvest and enumeration, passage to new culture flasks was performed. The process was repeated until a 50% decrease in the harvested total cell counts was recorded, based on the average cell counts at passage levels 2–5. Based on the obtained results, the primary cells were used for experimentation up to passage levels where a decrease of ≤25% in the harvested total cell counts was recorded, based on the average cell counts at passage levels 2–5. 

### 2.4. Primary ASC-Type Qualification: Adipogenesis Assays

In order to verify the presence of stem cell properties in the established ASC types, chemical adipogenesis assays were performed. Cellular differentiation was evaluated by lipid droplet deposition, Oil Red O (ORO) staining of the droplets, and ORO colorimetric quantification.

#### 2.4.1. ASC Expansion and Adipogenic Induction

Several WCB II vials of ASCs were thawed and the cells were seeded at 10^3^ cells/cm^2^ in 12-well culture plates. The cells were expanded to sub-confluency levels (i.e., 50–80%) in CfM. Then, the chemical induction was initiated by culturing the cells in adipogenic medium (AM). Three different AM formulas were parallelly used, where the basal medium (i.e., DMEM, ITS 1×, 100 μM indomethacin, 1 μM dexamethasone, and 100 μM IBMX [Sigma Aldrich, Buchs, Switzerland]) was used directly or was supplemented with 5% HPL or with 10% FBS. Appropriate control plates were prepared and maintained. The media were exchanged twice per week during the adipogenic differentiation process. For the induced cells, the differentiation phase was stopped after 14 days and endpoint readouts were investigated. 

#### 2.4.2. Induced ASC Lipid Droplet Analysis: Oil Red O Staining and Quantification

Following adipogenic induction, the adherent cells were rinsed with PBS and were fixed for 10 min at ambient temperature in the 12-well plates using 4% PAF (Sigma Aldrich, Buchs, Switzerland). The wells were then washed twice with deionized water. Volumes of 1 mL of working solution (0.18%) of Oil Red O (Sigma Aldrich, Buchs, Switzerland) were added to the wells and the plates were incubated at ambient temperature with slow agitation for 15 min. The wells were then washed twice with deionized water and the plates were stored at 4 °C until imaging, with the cells immerged in deionized water. Imaging was performed at 10× magnification using an Olympus IX81 microscope (Olympus Corporation, Tokyo, Japan). The level of lipid droplet formation was recorded based on tri-operator microscopic assessments and by quantification of the captive Oil Red O by colorimetry.

### 2.5. Cytotoxicity Evaluation of Biocidal and Antifungal Compounds

In order to validate the use of the established primary cell sources in cytotoxicity assays, several compounds of interest for topical application in burn care were investigated. Therefore, primary cell sources and reference cell lines were comparatively used for in vitro cytotoxicity assessments, as methodologically described point-by-point in Section 2.5.1, Section 2.5.2, Section 2.5.3 and Section 2.5.4.

#### 2.5.1. Biocides and Antifungal Compound Sources

Using the established primary cell sources, two commercial formulations of HOCl (i.e., Vashe^®^, 0.033% HOCl, Urgo Medical, Chenôve, France; Brio^®^, 0.025% HOCl, Briotech, Everett, WA, USA) were assessed for in vitro cytotoxicity and were compared to CLX 0.10% (CHUV Pharmacy, Lausanne, Switzerland). Importantly, HOCl was retained for this study as it was previously reported to be safer than CLX. Of note, Vashe^®^ can be used in the USA as a standard of care but not in Europe because the product does not have the medical device status (i.e., CE mark). 

An investigational antifungal drug (i.e., the AR-12 compound, University of Iowa) was retained for the study and was assessed for in vitro cytotoxicity using the established primary cell sources. The small-molecule AR-12 has a broad spectrum of activity, making it a potential effective treatment against yeasts (e.g., *Candida albicans*), molds (e.g., *Fusarium* spp.), and dimorphic fungi (e.g., *Blastomyces* spp.). The AR-12 compound was obtained in crystal-like white powder form and was solubilized in DMSO at 10 mg/mL, with storage at −20 °C before use. 

#### 2.5.2. Sample Preparation for In Vitro Cytotoxicity Assays

All of the samples were prepared on the day of the assays. Each product/stock solution was diluted with CfM or CAM until the desired concentration ranges were obtained for the in vitro cytotoxicity assessments, based on OECD recommendations (i.e., with serial dilutions) [14,15]. The tested CLX- and HOCl-based biocides were used as received, apart from dilution with cell culture medium. 

#### 2.5.3. Validation of the Cytotoxicity Model in 24-Well Plates

To perform the cytotoxicity assays, WCB II vials of primary cells and vials of control cell lines (i.e., HaCaT keratinocytes) were thawed and were expanded to confluency. The cells were then harvested and seeded in 24-well plates at densities of 3 × 10^3^ and 6 × 10^3^ cells/well. The cultures were maintained for 7 days as described previously. The test items were then dispensed in the wells of the culture plates, following dilution in CfM or CAM. The Vashe^®^ source was tested for cytotoxicity at final HOCl concentrations of 786–6290 µM. The Brio^®^ source was tested for cytotoxicity at final HOCl concentrations of 596–4766 µM. The CLX source was tested for cytotoxicity at final concentrations of 0.2–1978 µM. Regarding the AR-12 antifungal compound, cytotoxicity was assessed for a concentration range of 0.27–139 µM. The retained test item experimental concentrations were selected based on the OECD 442D guideline and on high-throughput screening principles [15,20]. 

After 48 h of cell treatment with the test items, the solutions were removed and a 10% solution of CellTiter^®^ (Promega, Madison, WI, USA) in CfM or CAM was added in every well for assessment of cellular viability. The plates were then placed in the incubator for 90 min. The solutions were transferred to a 96-well plate and were analyzed on a Varioskan LUX 3020 spectrophotometer (ThermoFisher Scientific, Waltham, MA, USA) at 490 nm with agitation. 

#### 2.5.4. Cytotoxicity Model Optimization in 96-Well Plates

Following the cytotoxicity model validation in 24-well plates, the same procedure was used for optimization and upscaling in 96-well plates. The culture plates were seeded with 5 × 10^3^ cells/well (i.e., for primary cells) and 10^4^ cells/well (i.e., for HaCaT keratinocytes) in a CfM volume of 100 µL per well. Specifically, the cell seeding density was optimized to obtain at least 50% of monolayer confluency in 24 h of culture. The cells were expanded for 24 h and were then treated with the test items for 48 h before endpoint measurement of viability was performed with the CellTiter^®^ assay. CfM was used as a positive control and PBS as a negative control. Each assay was performed in triplicate, of which every independent repeat was conducted on a different day, with fresh stock solutions and cells that were independently collected. 

### 2.6. Statistical Analysis and Data Presentation

For cell viability and toxicity studies, all of the obtained datasets were analyzed using Microsoft Office Excel^®^ (Microsoft Corporation, Redmond, WA, USA) to assess cell viability and were converted into percentages of the control, with standard deviations plotted as error bars. Data calculations and/or presentations were performed using the GraphPad Prism^®^ software version 8.3.0 (GraphPad Software, San Diego, CA, USA) or Microsoft Office Powerpoint^®^ (Microsoft Corporation, Redmond, WA, USA).

## 3. Results

### 3.1. Primary Cell Bank Establishment and Cell Source Sustainability Evaluation

**Figure 1:**
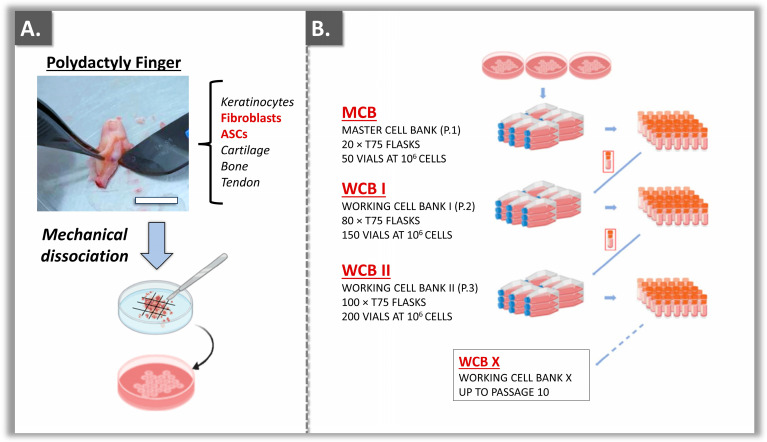
Schematic illustration of the culture initiation phase and tiered primary cell bank establishment for the primary dermal fibroblast and ASC sources from polydactyly digits. (**A**) Tissue procurement and adherent cell culture initiation phase. Scale bar = 5 mm. (**B**) Multi-tiered primary cell banking workflow, with mean cell bank lot size. Each established primary cell type was used to derive progeny MCB and WCB lots. ASC, adipose-derived stem cells; MCB, master cell bank; P, passage level; WCB, working cell bank.

In order to prepare sustainable sources of primary cells, tissue explant cultures and subsequent serial in vitro cell expansions were performed for each dermal fibroblast and ASC source (i.e., four polydactyly donors included in total, Figure S1). One donor was excluded from the study, as the tissue procurement phase was exceedingly lengthy (Figure S2). All the tissues were processed and enabled to prepare donor-specific MCB lots and two WCB lots of an appropriate size (Figure 1).

It was noted that despite the small size of the starting biological materials, systematic parallel isolation of dermal tissues and hypodermal fat was possible (Figure 1 and Figure S2). All cell bank lots were prepared and analyzed for conformity based on pre-determined and homogeneous criteria (Table 1).

**Figure 2:**
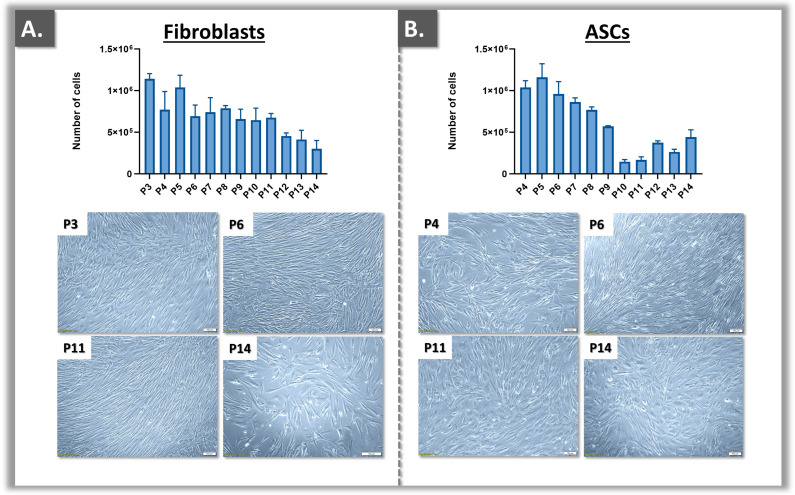
Primary cell proliferation data over serial in vitro passages (i.e., passage levels 3–14) for polydactyly-derived dermal fibroblasts (**A**) and adipose-derived stem cells (ASC; (**B**)). Experiments were performed in triplicate and standard deviations were presented around mean values. Scale bars = 100 µm.

All of the cryopreserved materials were assessed as being compliant with the applicable controls and the related acceptance criteria (Table 1). Specifically, the considered primary fibroblasts were qualified as such based on several predetermined attributes (i.e., morphological, behavioral, and proliferative aspects of the cultured cells; Table 1), exclusion criteria (i.e., absence of cell population contamination), and manufacturing process-related parameters (i.e., specific dissection of dermal tissue, use of specific fibroblast proliferation medium). Namely, any potential contamination of the fibroblast populations by keratinocytes or ASCs was technically excluded by the inability of such contaminants to sustainably proliferate in CfM and internal in-process controls confirmed the absence of contamination (Table 1). Following the preparation of the primary cell banks, the materials were investigated in more detail for the EOP determination (i.e., fibroblasts and ASCs). Therein, dermal fibroblasts displayed higher proliferation potential under standardized culture technical specifications (i.e., 1500 cells/cm^2^ at seeding, 7 days of growth; Figure 2).

Specifically, dermal fibroblasts slowly declined over passages in the cell proliferation assays and could be used for experimentation purposes up to passage levels 10–11 (Figure 2A). However, a more rapid decline in proliferation over passages was recorded for ASC cultures, which could be used for experimentation purposes up to passage levels 7–8 (Figure 2B). Overall, given that WCB II lots were available at passage level 3 and that at least three more passages could be used for cell banking purposes, it was confirmed that several million assays could technically be performed using the same primary cell type (Figure 1 and Figure S1). In detail, for an estimate yield of 50 MCB vials, 150 WCB I vials, and 200 WCB II vials, complete expansion of the available cell stocks would yield 1.5 × 10^6^ WCB II vials (i.e., 50 × 150 × 200 vials; Figure 1). Importantly, the sustainability of the described primary cell banks requires the consistent use of a defined passage level for in vitro cytotoxicity assays (e.g., passage level 4 or 5). Therein, the retained passage level should be within the validated in vitro cell lifespan, as determined during the EOP studies (Figure 2).

### 3.2. Functional Qualification of Polydactyly ASC Sources

**Figure 3:**
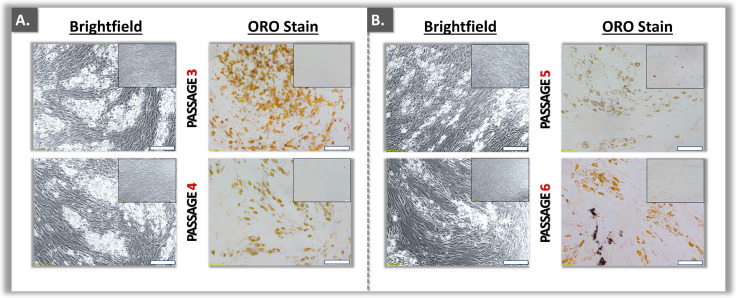
Adipogenesis induction assays in serial subcultures (i.e., passage levels 3 and 4 (**A**), 5 and 6 (**B**)) of polydactyly-derived ASCs. Unstained lipid droplets and ORO-stained lipid droplets were consistently observed throughout the investigated passage levels. Assay development data are presented in Figures S3 and S4. Scale bars = 200 μm. ASC, adipose-derived stem cells; ORO, Oil Red O.

In order to confirm the stem cell nature of the prepared polydactyly ASC sources, adipogenesis studies were performed. Preliminary assessments indicated that the use of adipogenic medium containing FBS resulted in enhanced levels of lipid droplet production by the cells as compared to the use of HPL (Figure S3). Several WCB I and WCB II lots of primary ASCs were then tested for functionality in adipogenic differentiation, using Oil Red O staining and subsequent dye quantification. Therein, lipid droplet accumulation was consistently observed at least until passage level 6 (Figure 3).

Specifically, adipogenesis-related markers (i.e., intra-cellular lipid droplets) were found to be significantly present at least until passage level 6 (Figure 3, Table 2).

Specifically, the significant presence of adipogenesis markers was evidenced for the cells induced in FBS-based medium (Table 2). Notably, the presence of these markers was systematically less pronounced in HPL-based medium (Table 2). Overall, based on the adipogenesis differentiation data at the cellular level, an optimal adipogenic protocol was devised (i.e., 10% FBS medium, 80–100% confluency level at induction initiation, Figure S3). Generally, while ASCs were able to proliferate up to passage levels 7–8, the functional qualification assays restricted their further experimental use to passage levels 3–6 (Figure 2 and Figure 3, Table 2). Overall, the four ASC sources included in the study were validated in terms of adipogenesis potential. 

### 3.3. Cytotoxicity Model Validation Using Primary Polydactyly-Derived Dermal Fibroblasts

In order to obtain in vitro cutaneous cytotoxicity data, the experimental testing conditions were first established in a 24-well plate setup. Specifically, these conditions enabled us to microscopically monitor the morphology of the target cells throughout the proliferation phase and the incubation period with the test items. For the assays, various dilutions of the biocidal and antifungal products of interest were used, as reported in Table 3.

**Figure 4:**
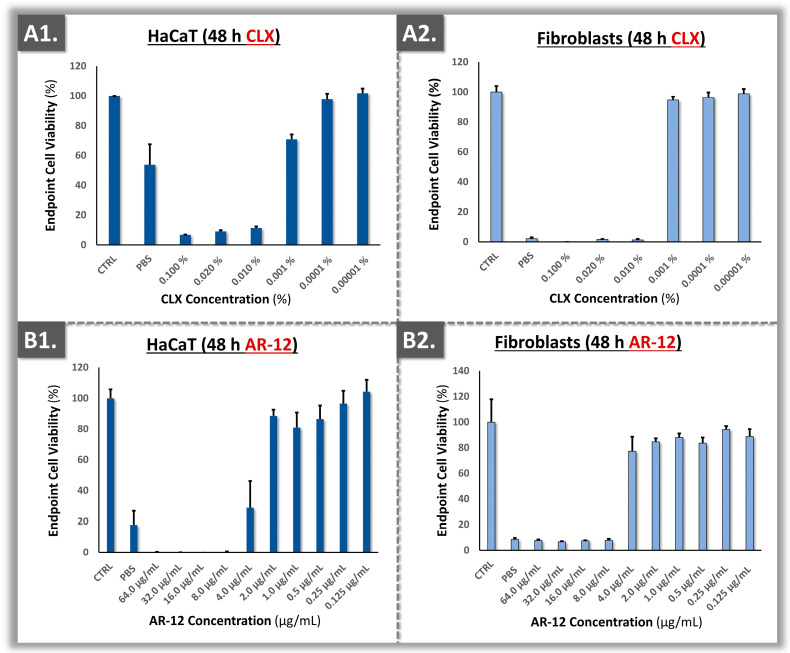
Cytotoxicity results for HaCaT keratinocytes and polydactyly-derived primary dermal fibroblasts exposed to the test items. (**A1**,**A2**) Endpoint viability of the target cell populations after exposure to CLX. (**B1**,**B2**) Endpoint viability of the target cell populations after exposure to AR-12. CLX, chlorhexidine.

**Figure 5:**
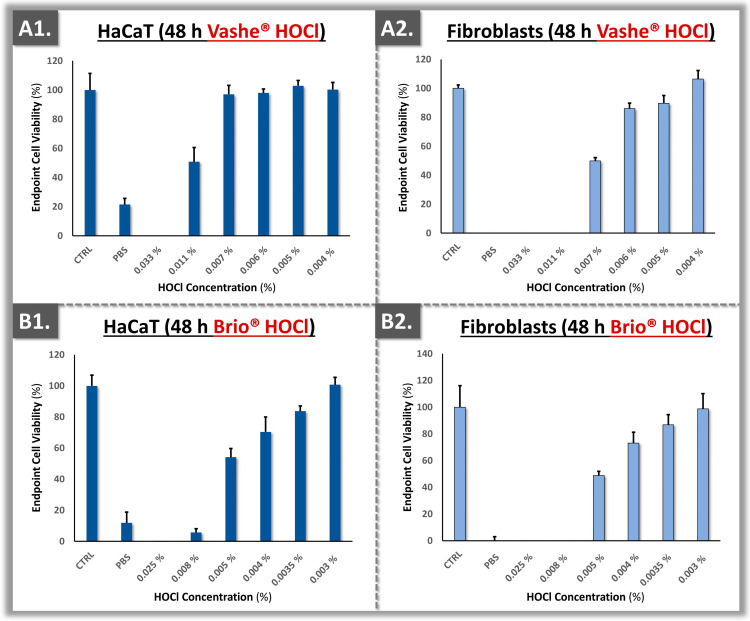
Cytotoxicity results for HaCaT keratinocytes and polydactyly-derived primary dermal fibroblasts exposed to the test items. (**A1**,**A2**) Endpoint viability of the target cell populations after exposure to Vashe^®^ HOCl. (**B1**,**B2**) Endpoint viability of the target cell populations after exposure to Brio^®^ HOCl.

Once all the technical specifications were determined, the assays were transposed into 96-well plates for experimental throughput optimization. Therein, the polydactyly-derived primary dermal fibroblasts were directly compared to immortal HaCaT keratinocytes in terms of sensitivity to CLX, to two HOCl sources, and to AR-12 (Figure 4 and Figure 5). 

Methodologically, HaCaT cells were retained for the in vitro analysis of topical compounds, as keratinocytes are major constituents of the epidermis and are recommended by the OECD for skin sensitivity testing [15,17]. In parallel, primary dermal fibroblasts were retained for the assays, as these cells are highly prevalent in the wound bed (i.e., in the dermis) of cutaneous burns, which is exposed to the environment and to topical treatments in hospitalized patients. Despite the fact that infant fibroblasts may differ from adult fibroblasts (i.e., for in vitro cellular models), they were assessed as being more relevant from a species point-of-view than transformed 3T3 mouse fibroblasts for example. 

The obtained results showed that the interaction of Brio^®^ HOCl with the primary dermal fibroblasts resulted in more sensitivity than that observed in the Vashe^®^ HOCl group (Figure 5). In the case of CLX, the primary dermal fibroblasts displayed similar cytotoxicity profiles with HaCaT keratinocytes, with a similar dose-dependent response (Figure 4(A1,A2)). Finally, a dose-dependent response was also observed for the AR-12 antifungal compound (Figure 4(B1,B2)). Overall, a trend of lower endpoint viability of the primary dermal fibroblasts was evidenced as compared to the HaCaT keratinocytes, suggesting that the primary cells were more sensitive to topical compound cytotoxicity in the retained experimental setup (Figure 4 and Figure 5). 

### 3.4. Cytotoxicity Model Validation Using Primary Polydactyly ASCs

**Figure 6:**
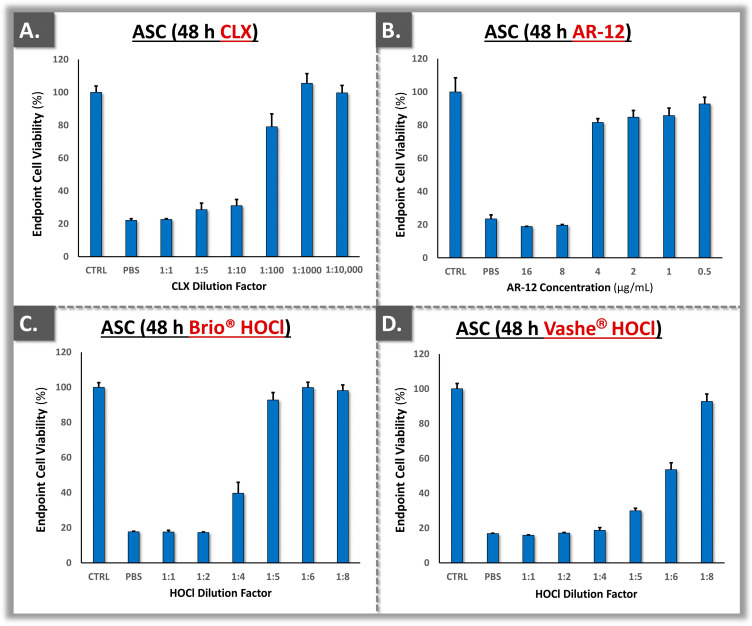
Cytotoxicity results for polydactyly-derived ASCs exposed to the test items. (**A**) Endpoint viability of the target cell populations after exposure to CLX. (**B**) Endpoint viability of the target cell populations after exposure to AR-12. (**C**) Endpoint viability of the target cell populations after exposure to Brio^®^ HOCl. (**D**) Endpoint viability of the target cell populations after exposure to Vashe^®^ HOCl. ASC, adipose-derived stem cells; CLX, chlorhexidine.

Polydactyly-derived ASCs were included in the topical cytotoxicity model, as hypodermal tissues are often exposed in third degree burns. Specifically, such cell sources were previously proposed for complementary in vitro cytotoxicity testing and were considered herein to be relevant in a severe burn patient treatment regimen [19]. Following ASC source functional validation, the cells were tested for cytotoxicity against CLX, two HOCl sources, and AR-12 (Figure 6).

Generally, the ASCs presented similar cytotoxicity profiles to the primary dermal fibroblasts in the established in vitro model (Figure 4, Figure 5 and Figure 6). In addition to the clinical relevance of studying ASCs for topical cytotoxicity evaluation in burn care, the standardized cell source establishment and functional validation schemes presented herein contributed to the methodological robustness of the reported datasets. Of note, the experimental cytotoxicity data in ASCs were expressed in dilutions of the finished products that are available for burn wound management (Figure 6). Specifically, such results enabled us to consider the limitations of the in vitro cell-based model as a whole, as the undiluted products are safely clinically used in humans, despite showing some strong reductions in cell viability in vitro (Figure 6). 

From a product formulation viewpoint, it was noted that several excipients and solvents are used to stabilize and dilute the CLX and HOCl active component. Such additives (e.g., alcohol in the case of CLX) may contribute to the global effects of the finished products on cell cultures. In the presented experiments, a low concentration of CLX (i.e., 0.10%, alcohol-free variant) was used and it was diluted between 10- and 100-fold in order to avoid toxicity in cultured cells (Figure 4 and Figure 6). Therefore, high attention must be paid to the concentration of the stock CLX solutions which are available for burn centers, as CLX preparations can vary significantly [8]. More recently, the Vashe^®^ product has been clinically used in the USA with very high safety for wounds and burns. In the present study, the two investigated HOCl formulations displayed similar in vitro toxicity at the same dilutions on the primary cell cultures (Figure 5 and Figure 6). Finally, similar cytotoxicity values (i.e., 4–8 µg/mL) were recorded for primary dermal fibroblasts and ASCs for the AR-12 compound, confirming the applicability of such cell types to investigate potential novel antifungal treatments in screening assays (Figure 4 and Figure 6). 

## 4. Discussion

### 4.1. Available Cellular Models for In Vitro Topical Compound Cytotoxicity Assays

Cytotoxicity assays play a major role in fundamental research and in drug discovery. Notably, such tools are useful for screening chemical libraries for toxic compounds or for evaluating the preclinical safety of a newly developed chemical entity before going into later stages of drug development. Additionally, these assays may provide important in-process controls to monitor the cellular toxicity of marketed drugs and devices (e.g., topical antifungals, biocides), as well as changes in product formulations [6,17]. As previously mentioned, various transformed animal cell lines (e.g., 3T3, CHO cells) are recognized by several normative organizations and pharmacopoeias as reference cell substrates, yet their biological and physiological relevance are sub-optimal in a human clinical context [15,16,17]. While HEK or human embryonic stem cells (hESCs) are more relevant from a species standpoint, the physiological relevance with post-natal cutaneous structures is also considered sub-optimal. Such considerations are specifically presented in the context of burn wound clinical treatments, where the cutaneous cellular components are already submitted to high levels of multi-factorial stress. 

In terms of skin cell models, HaCaT keratinocytes have been notably used as standard substrates [6,15,18]. HaCaT cells consist of spontaneously immortalized human keratinocytes that are widely used in research, notably in the investigation of skin irritation, skin cancers, topical cytotoxicity, and other skin-related issues [18,24,34]. This is made possible by the numerous advantages that this cell line presents, such as unlimited proliferation capability, undemanding culture conditions, and the retention of differentiation capacity [23,24]. Furthermore, this cell line is considered as relevant for use in laboratory skin models for screening purposes, since keratinocytes are one of the most prominent cell types in human epidermal tissues. Specifically, being one of the first cell types of the skin to be exposed to environmental components, keratinocytes are in the OECD recommendations for the in vitro prediction of skin sensitization by chemical products [15]. Parallelly, 3T3 mouse embryonic fibroblasts and NHK keratinocytes are recommended by the OECD for in vitro cytotoxicity testing and for the estimation of starting oral dosages for acute oral systemic toxicity [14].

Overall, the use of continuous cell lines for in vitro testing purposes is driven by the sustainability and homogeneity of the specified cell sources. Due to the need for enhanced clinical and physiological relevance of the retained models, it was of interest to develop alternative models using stem cells or primary cell sources that can be standardized [18,22]. Importantly, cellular proliferation characteristics and the in vitro lifespan of cell cultures are dependent on the method of cell type establishment from the biopsy and on the subsequent culture conditions [35]. Of note, it was reported that the factors that had the highest impact were the cell seeding density and oxygen concentration, although nutrient media, donor age, and anatomical site of the biopsies also determined the longevity of the in vitro cell culture potential [35]. Thus, based on previous reports and on the sustainable primary cell source management strategy presented herein, polydactyly-derived cellular substrates were determined to bare high potential for application in topical compounds in vitro cytotoxicity assessments. 

### 4.2. Suboptimal Safety Profile of CLX as a Topical Antiseptic in Burn Care

Due to the vulnerable nature of burn patients, it is of high importance to thoroughly assess the cellular toxicity potential of the products and preparations that are clinically used on a large scale. Therein, excessive inherent product toxicity could produce drastic detrimental effects [4,36]. In burn patient care, the initial use of cleansers and biocides has notably gained much interest to decrease the colonization and infection risks for the treated patients. From this standpoint, the gold standard in topical disinfectants has been CLX, with hundreds of thousands of liters used annually for disinfection in a given university hospital setting [8]. 

Of note, CLX is a cationic polybiguanide used as a disinfectant and antiseptic agent with a broad spectrum of activity against Gram + and − bacteria, yeasts, and aerobes or facultative anaerobes. It is a positively charged and lipophilic molecule that interacts with the phospholipids and lipopolysaccharides of the bacterial membrane and disturbs its osmotic equilibrium. This in turn allows for CLX to penetrate the bacterium and precipitate its cytoplasmic contents, leading to cell death [8,10,37]. In the past, CLX has been used in various concentrations and with various excipients in intensive care, general surgery, and in burn units around the world [8,38]. Despite its documented efficacy, CLX is no longer in the primary recommendations for open wound cleansing, due to potential damage to patient tissues [4,6,7,8,9,10,11]. Therefore, there is an acute clinical need for alternative biocides presenting better cytotoxicity profiles as compared to CLX, wherein HOCl has been assessed as highly promising [12,13,39,40,41]. 

Methodologically, the selection of CLX and HOCl was thus justified in the present study for comparative evaluations of a potential replacement of the gold standard CLX. Specifically, earlier studies and surveys conducted in major burn centers have confirmed that CLX had been the reference antibacterial compound [8]. Notwithstanding, burn centers continuously strive for the amelioration of quality and the minimization of risks for topical products that are used on burn patients, as they represent a vulnerable population. Thus, despite its current commercial unavailability in Europe and Switzerland, HOCl was considered to be of particular interest for instances where large quantities of antibacterials are applied (e.g., hydrotherapy for severe burn patients).

### 4.3. Topical HOCl Constitutes a Safer Alternative to CLX in Burn Care

Recent clinical recommendations were made available for the topical human use of HOCl as a biocide [12,13]. Despite its commercial availability in North America, HOCl is considered as a new chemical entity in Switzerland and in Europe and must therefore be assessed prior to being brought on the market for human topical use [16]. Interestingly, over the past 100 years, solutions of sodium hypochlorite (NaOCl, Dakin’s solution; 0.125–0.5%) have been and are still used in wound care and in various skin conditions [42,43]. Alongside medical honey, hydrogen peroxide, or iodine, NaOCl has benefited from long-term clinical use in multiple indications [4,44]. More recently, the advancement in formulation methods has provided electrochemical-based HOCl solutions, which are of high interest due to their overall safety, stability, and efficacy. Specifically, such powerful oxidizing solutions were extensively studied in the search for inexpensive and non-toxic disinfectants covering microbial, fungal, and viral pathogens [45,46,47].

Of note, HOCl is a physiological endogenous compound that is produced by the immune system, which can lead to cell death by a decreased uptake of nutrients, inhibition of protein synthesis, decreased oxygen uptake, and by various means of DNA alteration. Low HOCl concentrations of 200 ppm can be very effective in decontamination with appropriate contact times. Optimal biosafety was reported for antimicrobial doses of HOCl, with use at 100–200 ppm in the eyes or in intraperitoneal wound care [48]. Such results were interpreted positively from an in-use viewpoint, as the in vitro data gathered herein showed cytotoxicity at doses 50 times lower (i.e., 4 μg/mL). Such results highlighted the significant difference in sensitivity between the controlled in vitro setups and in vivo use. In European countries, there is a progressive movement to accept HOCl as a biocide for skin contact, with Finland, Sweden, Denmark, Austria, France, Portugal, and Poland accepting registrations more rapidly. In Switzerland, HOCl can already be used for surface disinfection, but its use on human or animal skin would require the registration of a product as a medical device.

Generally, the in vitro cytotoxicity results presented herein have confirmed that HOCl exhibits less toxicity than CLX (Figure 4, Figure 5 and Figure 6). The findings were in alignment with those of Ortega-Peña et al., where the authors compared the cytotoxicity attributes of various biocides using human skin fibroblasts [40]. Therein, the authors demonstrated that in addition to the limited cytotoxic activity of HOCl, it could also have a stimulatory effect on fibroblast and keratinocyte proliferation [40]. In the primary cell-based models reported herein, quantitative differences were outlined in terms of cytotoxicity between the two HOCl sources (Figure 5 and Figure 6). This might be due to different fabrication methods of the active compound and formulation specifications of the finished product [12,49]. Specifically, while Brio^®^ consists of diluted and pure HOCl, Vashe^®^ also contains NaCl [50,51]. In detail, HOCl is naturally unstable; thus, in order to maximize the stability of the solution, the formulation should contain as low as possible concentrations of various compounds or ions [12].

Overall, it was observed that HOCl was much less toxic for human primary cells than CLX, as previously reported (Figure 4, Figure 5 and Figure 6) [39]. In general, 0.05% CLX has been used routinely in burn care [8]. Previous surveys indicated that CLX is the most widely used biocide and that the quantities for use in disinfection are very high in Swiss hospitals [8]. Unfortunately, there has been high variability in the formulations and dosages of CLX that are commercially available. Therein, some CLX formulations contain alcohol, which could bare negative effects when used in undiluted form on burn patients. Therefore, it is very important that the correct formulation and dosage of CLX are available for the burn center [8]. Generally, lowering patient exposure to chemical agents showing toxicity issues would be of tangible benefit, particularly for highly vulnerable populations such as burn patients.

### 4.4. Primary Cells for Highly Sustainable and Biologically Relevant In Vitro Cytotoxicity Assays

The sustainability of the primary cell sources established for this study was confirmed based on the validation of the respective cryopreserved cell stocks (Figure 1, Table 1). Therein, the polydactyly-derived dermal fibroblasts and ASCs could be sustainably banked, with a recorded higher proliferative potential (i.e., in terms of in vitro lifespan) for the former cell types (Figure 2). The primary nature of the established cell sources and their correspondence with relevant anatomical sites in burn wounds (i.e., dermal fibroblasts for the dermis, ASCs for the hypodermis) then contributed to the enhanced biological relevance of the presented in vitro models. Specifically, cytotoxicity testing using primary cells contributes to better approximation of the use of biocides on the compromised and fragile cutaneous tissues of burn patients (i.e., more transposable results than models using immortal cells).

From a quantitative perspective, comparable cytotoxicity cut-off values were experimentally obtained between the HaCaT cell line and the primary cells (Figure 4 and Figure 5). Thus, the developed in vitro model using primary cells could be used alternatively or as a complement to classical cell-based assays, particularly for topical pharmaceuticals. Such results contrasted with previous reports, which compared primary fibroblasts to continuous 3T3 murine fibroblasts [22]. Therein, the authors tested 26 reference chemicals and were able to show that 3T3 cells were generally more sensitive to the investigated chemicals than the human fibroblasts [22]. However and importantly, a limiting factor of the referenced study was the lack of methodological standardization on the substrate side, as primary fibroblasts were purchased from a cell collection and no passage level number was indicated [22]. By standardizing this aspect further in the present study (i.e., use of defined and consistent passage levels for the in vitro assays), the sustainability and robustness of the experimental setup were augmented. 

From a technical standpoint, relatively low cell seeding densities could be retained when developing alternative standardized assays to ensure limited impacts on cell viability and cytotoxicity responses. This approach may lead to cost-effective assays, due to the sparing use of biological materials. Furthermore, cell densities should be standardized to the type of culture vessel that is used in the assay. Specifically, the comparison of different cell confluency levels is important for the development of a standardized assay, to use the same number of cells for every experiment, and to avoid potential impacts/bias on the obtained results. Indeed, inappropriate confluency levels can have an impact on the obtained results during an assay and could lead to wrong conclusions being drawn on the possible cytotoxicity of a compound. 

### 4.5. Pertinence of Cell-Based Cytotoxicity Assay Readouts

In addition to the impact of the cell–substrate (i.e., cell lines or primary cell type) and the experimental technical specifications of the assay (e.g., cell seeding density) on the cytotoxicity results, test item- and readout-related considerations were brought forth. In detail, the OECD recommends the use of neutral red uptake (NRU) as a readout method to detect the viability of the cells for in vitro cytotoxicity assays [14,15]. While NRU is sensitive and cost-effective, this technique requires numerous handling steps (e.g., washing the cells, dye dissolving step) and requires changes in the culture conditions, which could have an impact on the cells [52]. For these reasons, the CellTiter^®^ method was chosen herein to determine cell viability. Of note, the CellTiter^®^ method does not quantify the lysosomal ability of viable cells to incorporate and bind the neutral red dye. Rather, it is a sensitive adenosine triphosphate (ATP) quantitative assay, which quantifies the ATP levels of the cells as a proxy for metabolic activity. Therefore, the obtained luminescence response is directly proportional to the number of living cells in culture in the well. The method can thus be used for high-throughput screening to indirectly characterize the cellular response following exposure to different compounds, with the advantage that it can be used in a single-step process.

### 4.6. Determination of Topical Cytotoxic Doses for the AR-12 Compound 

The AR-12 compound is a celecoxib analog, re-purposed (i.e., from oncology applications) as an antifungal because it was shown in vitro to work as a fungal ATP-competitive acetyl coenzyme A synthetase inhibitor, which could lead to autophagy and loss of cellular integrity [53,54]. The experimental results gathered herein enabled us to compare the obtained IC_50_ values of AR-12 to the minimum inhibitory concentration (MIC) of the molecule, which consists of the lowest dose at which the drug will inhibit the growth of a given microorganism. Against most medically relevant test strains of yeast, the reported MIC of AR-12 is 8 µM [53,54]. This value was close to the experimentally derived IC_50_ values of AR-12, which were 9.96 ± 0.35 µM and 10.71 ± 0.27 µM for HaCaT keratinocytes and primary dermal fibroblasts, respectively. 

Generally, it is important that the MIC of a compound does not exceed the IC_50_ concentration in cytotoxicity assays in order to be pharmacologically relevant. This ensures that the treatment provides a good risk–benefit balance for topical use, killing unwanted fungal species while remaining at a sub-toxic dosage for the patient tissues and cells. In the present case, the relatively high cytotoxicity of the AR-12 compound with respect to documented in vitro MIC values would practically restrict its use in yeast-based infections, due to the potentially incurred damages by the surrounding biological tissues. Of note, AR-12 was recently shown to also inhibit dihydroorotate dehydrogenase, a critical enzyme in the de novo nucleic acid biosynthesis in human cells [55]. This mechanism may partly explain the observed in vitro toxicity of AR-12 at the cellular level. Notwithstanding, such data should be put into perspective, as all of the investigated commercial products were shown to be cytotoxic herein in undiluted form, despite their clinical use in the same undiluted form (Figure 4, Figure 5 and Figure 6). Overall, from a product benchmarking viewpoint, the primary cell sources investigated herein were confirmed to constitute relevant targets for novel compound in vitro toxicity testing, as the sensitivity toward AR-12 was high for both primary dermal fibroblasts and ASCs.

### 4.7. Study Limitations and Future Perspectives

The main study limitations were related to the technical specifications used in the in vitro cytotoxicity assays and the low number of investigated biocides. Specifically, the retained cell seeding densities and confluency levels are determinant for cell viability and cytotoxicity responses. Varying the cell confluency levels in an assay can have an impact on the obtained results and could lead to wrong conclusions about the cytotoxicity attributes of a given compound. Therefore, further investigation of this parameter would be beneficial in order to augment the robustness of the experimental results. Furthermore, notwithstanding the known difficulties in the specific identification of primary dermal fibroblasts (i.e., absence of highly specific markers), their qualification could be further experimentally performed, notably for the assessment of identity and purity (e.g., p63 marking for exclusion of keratinocyte contamination) and specific functionality (e.g., vitamin C induction for confirmation of collagen production). Regarding the number of investigated biocides, it was designed to be low in the present study, due to the proof-of-concept nature of the in vitro cytotoxicity experiments. Specifically, the majority of the focus was set on the establishment and control of the primary cell sources to be used in the study, technically enabling further large-scale compound toxicity testing campaigns. Of note, alternative antiseptics and biocides of diverse chemical natures, as well as novel nano-biocides, may potentially be studied using the presented methodologies [56,57,58,59].

Thus, future perspectives based on the present study comprise the implementation of the established cellular model in screening assays for other antifungal analogs that could potentially be used topically for burn patients. While both hypotheses of the study were experimentally confirmed, several limitations to the generalizability and extrapolation potential of the presented results need to be set forth. Specifically, the sustainability of the established cell sources would need to be confirmed under real-world conditions, by using the constituted MCB/WCB stocks over several years, while validating the consistency of the obtained data on the long-term. Furthermore, comparative assays with alternative cutaneous cells (i.e., functionally specialized cells) should be performed in the context of in vitro cytotoxicity assays. Notably, the comparative use of adult patient cells would help to shed some light on the validity of infant polydactyly fibroblast models for approximating adult/elderly cutaneous tissues. Furthermore, the use of primary cell sources from donors of various age ranges would enable us to assess if a specific source is able to establish standardized and homogeneous cell stocks. Globally, as the validity of the presented cell banking model and the proof-of-concept application of the primary human cells in cytotoxicity assays were confirmed herein, the elements described hereabove may be considered for the design of future prospective investigations for topical in vitro cytotoxicity testing.

## 5. Conclusions

The present study set forth several methodological elements that enabled us to sustainably perform in vitro topical compound cytotoxicity assessments with enhanced biological relevance compared to models based on cell lines. Specifically, it was shown that primary cell sources derived from polydactyly tissues could be structured in tiered cell banks, with theoretical yields reaching millions of WCB vials following a single cell isolation procedure (i.e., 50–200 vials/batch, vials at passage levels 1–10 with 10^6^ cells/vial). Furthermore, it was discussed that primary dermal fibroblasts and ASCs were more anatomically and functionally relevant than continuous cell lines in the context of in vitro burn wound modeling. Therein, as topical treatments come into direct contact with multiple cell types in burn patients, the study retained primary cell sources from the dermis and hypodermis as substrates. Therefore, the established primary cell-based model was validated in cytotoxicity assays, focusing on biocidal and antifungal compounds of interest for burn wound care. In response to exposure to CLX, primary dermal fibroblasts displayed similar dose-dependent cytotoxicity than HaCaT keratinocytes. The results also revealed that the investigated primary cells were more sensitive to topical compound cytotoxicity than cell lines. Similar cytotoxicity values were obtained between two sources of HOCl and it was shown that HOCl was less cytotoxic than CLX when considering dilutions of ready-to-use products. Finally, similar cytotoxicity values (i.e., 4–8 µg/mL) were recorded for primary dermal fibroblasts and ASCs for the AR-12 compound. Generally, the present study confirmed the applicability of primary cell sources for standardized cytotoxicity assay development, despite the finite in vitro lifespan of primary cells. Overall, the study underscored the critical importance of thoroughly assessing topical compounds that are used or could be used in burn centers in order to continuously update and optimize the quality of the applied care regimens.

## Figures and Tables

**Table 1:** Control parameters for the prepared ASC and primary dermal fibroblast cell bank lots (i.e., in-process controls and post-process controls). ASC, adipose-derived stem cells; MCB, master cell bank; Ph. Eur., European Pharmacopoeia; WCB, working cell bank.

Parameters	Analysis Methods	Targets	Cumulative Acceptance Criteria ^1^	Application Levels	Application Timepoints ^2^
Cellular morphology	Microscopic operator assessment	Fibroblastic morphology	Fibroblastic morphology maintenance	MCB; WCB I; WCB II	In-process; before medium exchanges
Cell monolayer confluency level	Microscopic operator assessment	Endpoint confluency level 95–100%	Increasing confluency level throughout expansion; absolute value > 95% at harvest	MCB; WCB I; WCB II	In-process; before medium exchanges
Cell proliferation in culture	Microscopic operator assessment	At least 2.5 population doublings in expansion	At least 2.5 population doublings at harvest	MCB; WCB I; WCB II	In-process; after harvest
Cell monolayer homogeneity	Microscopic operator assessment	Monolayer homogeneity	Monolayer homogeneity maintenance	MCB; WCB I; WCB II	In-process; before medium exchanges
Cell population homogeneity	Microscopic operator assessment	No cell population contamination	Absence of a contaminant cell population	MCB; WCB I; WCB II	In-process; before medium exchanges
Cell proliferation medium aspect	Macroscopic operator assessment	No adventitious contamination	Absence of contamination indication (e.g., yellowing, turbidity)	MCB; WCB I; WCB II	In-process; before medium exchanges
Cellular viability at harvest	Manual enumeration with Trypan Blue dye	Harvest cell viability 85–100%	Viability level ≥ 85%	MCB; WCB I; WCB II	In-process; after harvest
Cellular viability at initiation from cryostorage	Manual enumeration with Trypan Blue dye	Initiation cell viability 85–100%	Viability level ≥ 85%	MCB; WCB I; WCB II	Post-process; after initiation from storage
Cellular recovery at initiation from cryostorage	Manual enumeration with Trypan Blue dye	Homogeneous cell bank lot (cell quantity/vial)	Cell quantity at filling ± 15% ^3^	MCB; WCB I; WCB II	Post-process; after initiation from storage

^1^ Acceptance criteria are applied to each individual culture vessel or each individual cell vial from a given lot. ^2^ In-process controls were performed during the cellular expansion phase leading to the preparation of the cell bank lot. Post-process controls were performed after the cryopreservation phase, at the time of vial thawing for further banking or experimental purposes. ^3^ Deviations were subjected to testing following Ph. Eur. chapter 2.9.40. Uniformity of dosage units.

**Table 2:** Effect of ASC passage level on the recorded presence of adipogenesis markers. Assessments were recorded as mean multi-operator gradings ^1^ at the end of the adipogenic induction phase. ASC, adipose-derived stem cells; FBS, fetal bovine serum; HPL, human platelet lysate; ORO, Oil Red O.

Passage Level	FBS-Based Medium		HPL-Based Medium	
	Lipid Droplet Density	ORO Quantification	Lipid Droplet Density	ORO Quantification
Passage level 3	+++	+++	++	++
Passage level 4	+++	+++	++	++
Passage level 5	+++	+++	++	++
Passage level 6	++	++	++	++

^1^ Gradings were attributed as follows for intra-cellular lipid droplets: (+++) = dense and homogeneous marker presence; (++) = scattered and homogeneous marker presence. Gradings were attributed as follows for ORO quantification: (+++) = ORO level within 80–100% of maximal values; (++) = ORO level within 60–79% of maximal values.

**Table 3:** Quantitative composition (i.e., active substance) of the various biocidal products used in the in vitro cytotoxicity assays.

Product	Original Product Concentration ^1^	Working Concentration Range
Chlorhexidine CHUV	0.100%	0.100–0.00001%
Brio^®^ HOCl	0.025%	0.025–0.003%
Vashe^®^ HOCl	0.033%	0.033–0.004%
AR-12	10 mg/mL stock solution	64.0–0.125 μg/mL

^1^ The AR-12 compound was not available in finished product form; thus, a stock powder form was used for the assays.

## Data Availability

The data presented in this study are openly available within the article files.

## Supplementary Materials

The following supporting information can be downloaded at: https://www.mdpi.com/article/10.3390/ebj5030024/s1, Figure S1: Schematic overview of the study methodology and primary cell banking model for cytotoxicity assessment; Figure S2: Photographic records of the procured polydactyly digits prior to cell isolation; Figure S3: Supplementary data for cellular adipogenesis assays; Figure S4: Supplementary data for cellular adipogenesis assays.

## List of Abbreviations

AM	Adipogenic medium
AR-12	Antifungal compound
ASC	Adipose-derived stem cells
ATCC	American Type Culture Collection
ATP	Adenosine triphosphate
CE	EU mark of conformity
CHO	Chinese hamster ovary
CHUV	Lausanne University Hospital
CLX	Chlorhexidine
DMEM	Dulbecco’s modified Eagle medium
DMSO	Dimethyl sulfoxide
DNA	Deoxyribonucleic acid
EOP	End-of-passage
EU	European Union
FBS	Fetal bovine serum
HaCaT	Immortalized human keratinocytes
HEK	Human embryonic kidney cells
hESC	Human embryonic stem cells
HOCl	Hypochlorous acid
HPL	Human platelet lysate
IBMX	3-isobutyl-1-methylxanthine
IC_50_	Dose inhibitory of one-half of the response
ISO	International standards organization
MCB	Master cell bank
MIC	Minimum inhibitory concentration
NRU	Neutral red uptake
OECD	Organization for Economic Co-operation and Development
ORO	Oil Red O
PAF	Paraformaldehyde
PBS	Phosphate-buffered saline
PCB	Parental cell bank
Ph. Eur.	European Pharmacopoeia
ROS	Reactive oxygen species
rpm	Rotations per minute
SD	Standard deviation
USA	United States of America
WCB	Working cell bank
3T3	Murine fibroblasts
