# Supplementary material for: Sustainable Primary Cell Banking for Topical Compound Cytotoxicity Assays: Protocol Validation on Novel Biocides and Antifungals for Optimized Burn Wound Care
Source: Eur Burn J. 2024 Aug 6;5(3):249–70. doi: 10.3390/ebj5030024 (PMC11544888; doi:10.3390/ebj5030024)
Supplement: Supplementary file 1 [file ebj-05-00024-s001.zip › ebj-3047160-Supplementary Materials.pdf]

european burn journal

MDPI

Supplementary Materials:

Sustainable Primary Cell Banking for Topical Compound Cytotoxicity Assays: Protocol Validation on Novel Biocides and Antifungals for Optimized Burn Wound Care

Zhifeng Liao, Nicolas Laurent, Nathalie Hirt-Burri, Corinne Scaletta, Philippe Abdel-Sayed, Wassim Raffoul, Shengkang Luo, Damian J. Krysan, Alexis Laurent * and Lee Ann Applegate *

1. Supplementary Figures

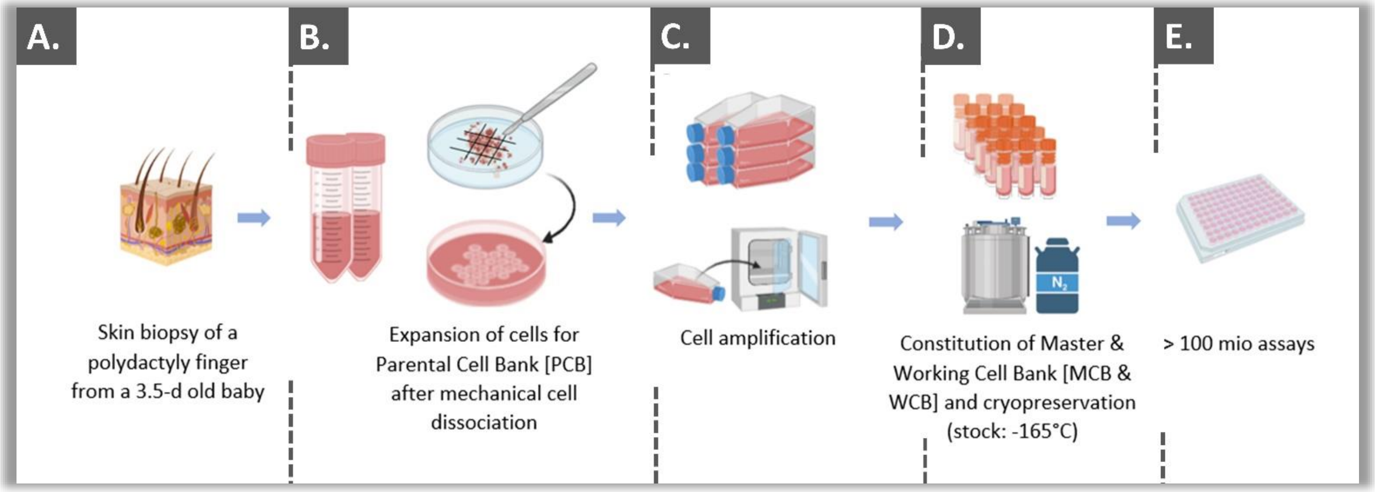

Figure S1. Schematic overview of study methodology and primary cell banking model for in vitro cytotoxicity assessments. (**A**) Tissue procurement phase (i.e., surgical waste). (**B**) Adherent cell culture initiation phase. (**C**) Serial cellular expansions for multi-tiered cell stock establishment. (**D**) Cell bank lot cryopreservation. (**E**) Initiation of WCB vials for cytotoxicity assays. MCB, master cell bank; PCB, parental cell bank; WCB, working cell bank.

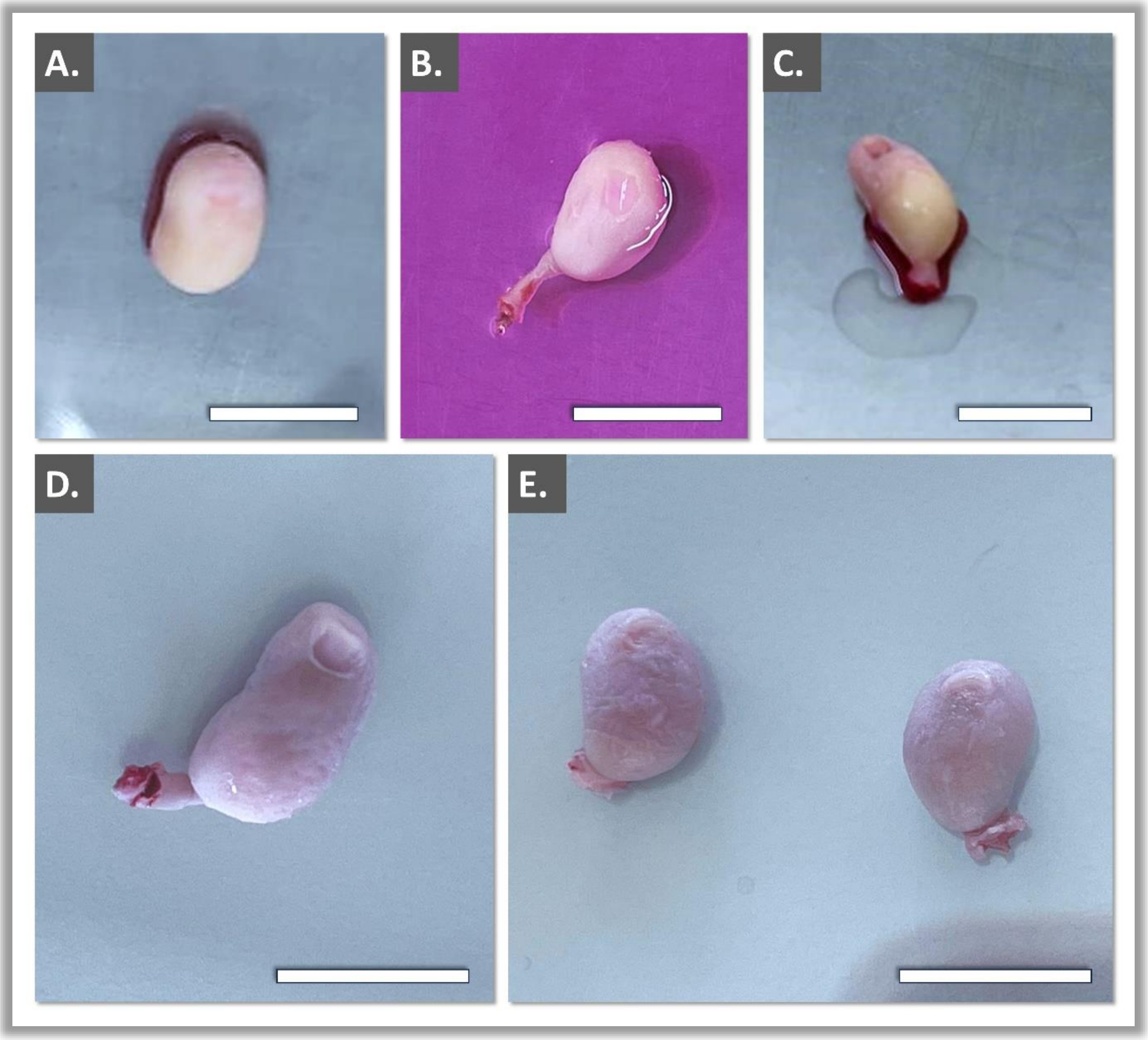

Figure S2. Photographic records of the procured polydactyly digits prior to primary cell isolation. (**A**) Polydactyly donor A. (**B**) Polydactyly donor B, which was excluded from the study due to non-conforming material procurement results. (**C**) Polydactyly donor C. (**D**) Polydactyly donor D. (**E**) Polydactyly donor E. Scale bars = 10 mm.

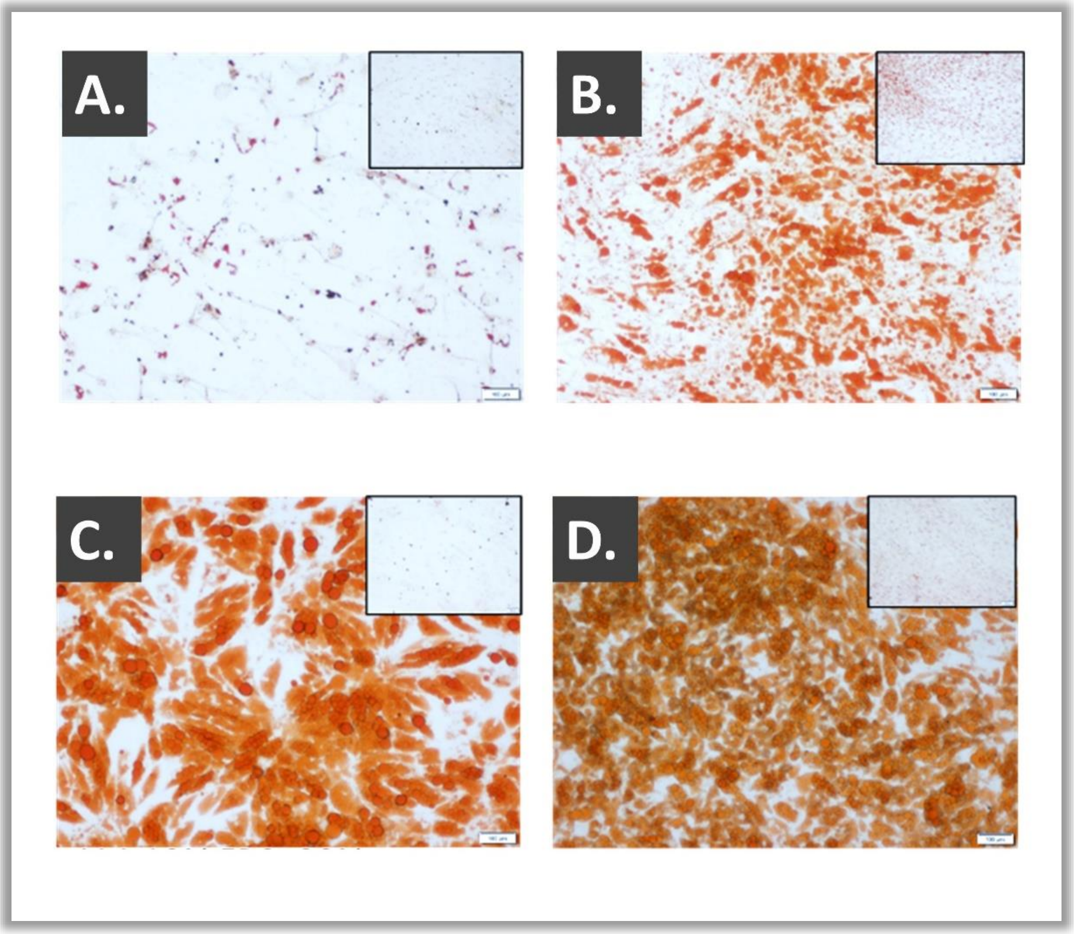

Figure S3. Adipogenic assay technical specification optimization results. Effects of various differentiation media compositions on ASCs (i.e., passage level 4), induced with AM-DMEM (**A**), AM (5% HPL; **B**), AM (10% FBS) 80% confluency (**C**), and AM (10% FBS) 100% confluency (**D**). Scale bars = 100–300 µm. Each condition was compared to its control, which did not receive AM (i.e., inserts in the upper right corners). Overall, the use of HPL was not very efficient for differentiation protocols when compared to those using FBS. As HPL has a cell growth stimulatory effect, it's use also complicated the protocol for differentiation, as the mechanism of cell growth is not stagnated immediately and both cell growth and differentiation occur simultaneously. The observation of lipid droplets is also less obvious with HPL than with a protocol using FBS. AM, adipogenic medium; ASC, adipose-derived stem cells; DMEM, Dulbecco's modified Eagle medium; FBS, fetal bovine serum; HPL, human platelet lysate.

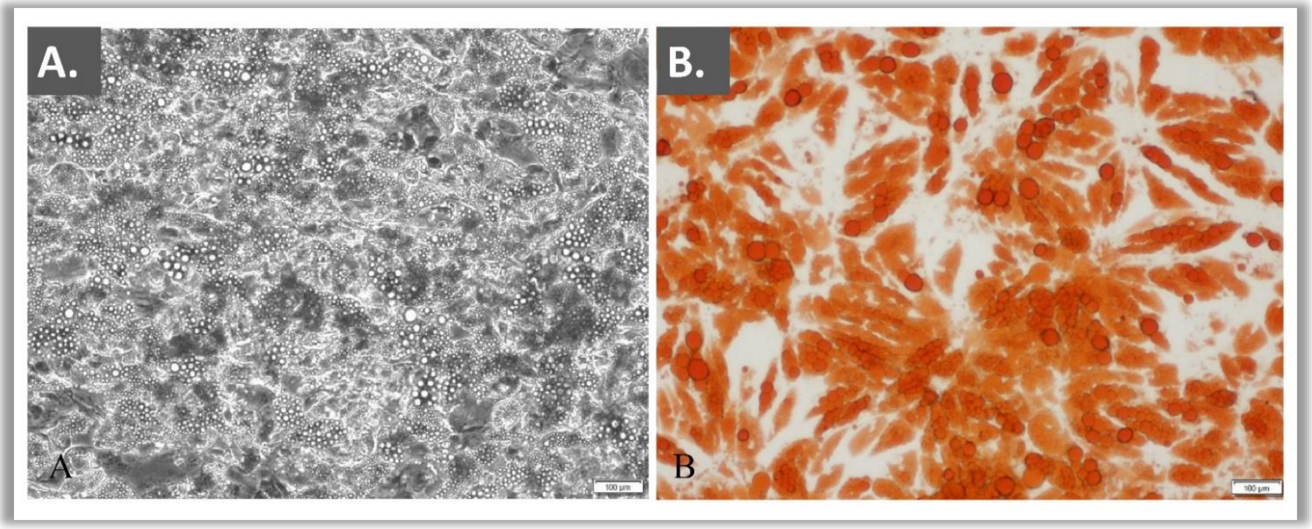

Figure S4. Lipid droplet formation in ASCs after 14 days of induction in AM (10% FBS) medium. The lipid droplets are noticeable before (**A**) and after (**B**) staining with Oil Red O. Scale bars = 100 µm. AM, adipogenic medium; ASC, adipose-derived stem cells; FBS, fetal bovine serum.
